# Pseudohypoparathyroidism Presenting with Ventricular Arrhythmia: A Case Report

**DOI:** 10.4274/Jcrpe.476

**Published:** 2012-03-08

**Authors:** Sedat Işıkay, İlyas Akdemir, Kutluhan Yılmaz

**Affiliations:** 1 Gaziantep University Faculty of Medicine, Department of Pediatrics, Gaziantep, Turkey; 2 Sema Hospital, Cardiologist, Istanbul, Turkey; +90 342 360 60 60/76476+90 342 360 39 28 dr.sedatisikay@hotmail.com

**Keywords:** Pseudohypoparathyroidism, arrhythmia, child

## Abstract

Pseudohypoparathyroidism (PHP) is a rare disorder characterized by varying degrees of unresponsiveness to parathyroid hormone. Patients usually present with hypocalcemia-induced seizures or tetany, whereas no case of hypocalcemia-induced cardiac arrhythmia in PHP has been described to date. In this paper, we report the case of a male adolescent with PHP type 1a who presented with hypocalcemia-induced ventricular extrasystoles (bigeminy, trigeminy) and mild corrected QT interval prolongation. The patient had brachydactyly and his second fingers and toes were longer than the others, a finding consistent with PHP. Laboratory tests detected hypomagnesemia, as well as elevated levels of creatine kinase and lactate dehydrogenase. Ventricular arrhythmia and abnormal laboratory tests improved with calcium supplementation and vitamin D treatment. The findings in this patient suggest that hypomagnesemia may make patients with PHP more susceptible to hypocalcemia and may thus prompt a state of hypocalcemia-induced arrhythmia or other cardiac complications. Key words: Pseudohypoparathyroidism, arrhythmia, child

**Conflict of interest:**None declared.

## INTRODUCTION

Pseudohypoparathyroidism (PHP) is characterized by hypocalcemia, hyperphosphatemia, and an elevated serum parathormone (PTH) level due to peripheral PTH resistance. Several subtypes are now recognized. In type 1, renal cAMP response to PTH is decreased because of a defect in G proteins. Some mutations in the alpha-subunit of G proteins have been demonstrated (1). G proteins are responsible for the coupling of surface receptors to activate adenylyl cyclase. PHP type 1 has variants, but generally, patients display distinctive clinical features such as a short stocky build, a round face, and brachydactyly. In spite of having these clinical features, some subjects have no biochemical abnormalities - these are cases of pseudoPHP. The mechanisms of genomic imprinting may elucidate the variations in PHP type 1 (1,2). In type 1b, the defect is confined to the kidneys, but it causes the same biochemical abnormalities. The patients do not exhibit any phenotypic changes. Patients with PHP usually present with tetany and seizures secondary to hypocalcemia. However, no case of PHP and hypocalcemia-induced ventricular arrhythmia has been described to date. In this paper, we report the case of an adolescent with PHP type 1a who presented with hypocalcemia-induced ventricular extrasystoles and mild corrected QT interval (QTc) prolongation.

## CASE REPORT

**Figure 1:**
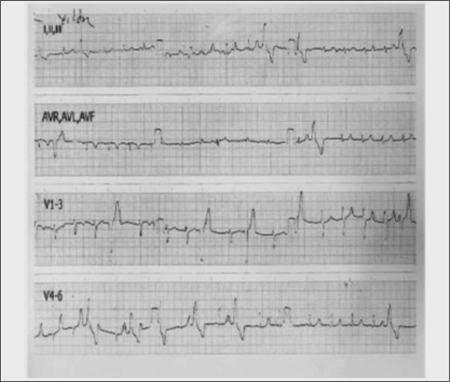
Electrocardiogram (25 mm/sec, 1mV/cm) showing ventriculararrhythmia and corrected QT interval (QTc) prolongation

**Figure 2:**
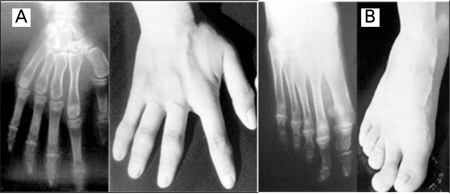
Brachydactyly due to short metacarpal/metatarsal bones andphalanges. Note that the second digits are relatively longer than theothers and less involved

A 15-year-old boy was admitted to hospital with the complaint of palpitations. His medical history revealed three generalized tonic-clonic seizures during the past year. He was the third child of nonconsanguineous healthy parents. No PHP-related findings were reported in first-degree relatives. Neuromotor milestones were within normal ranges. School performance was average. Weight and height were normal for age (25th-50th percentile for weight and 10th-25th percentile for height). Puberty level was Tanner stage IV. Mildly deformed fingers and toes were noted, and the second digits were longer than the others. In addition, he had lost all his teeth, excluding the mandibular incisors. His bone age was 14 years (Greulich-Pyle). An electrocardiogram revealed frequent ventricular extrasystoles (bigeminy and trigeminy; 12 beats/min, mean) with R-on-T phenomenon and mild QT prolongation (QTc: 0.47 sec) (Figure 1). Calcium (Ca) was 6.0 mg/dL (8.4-10.2); phosphorus, 10.6 mg/dL (2.7-4.7); alkaline phosphatase, 1074 IU/L (130-525); magnesium (Mg), 1.3 mg/dL (1.5-2.3); PTH, 750 pg/mL (10-65); creatine kinase (CK), 345 U/L (25-160); and lactate dehydrogenase (LDH), 303 U/L (117-230). Other biochemical analyses, including serum urea, sodium, albumin, blood bicarbonate, adrenocorticotropic hormone, thyroid stimulating hormone, total triiodothyronine, and total thyroxine were all normal. X-ray showed shorter metacarpal and metatarsal bones II and phalanges, with relative sparing of the seconds (Figures 2a and 2b). Computed tomography demonstrated a small intracranial calcification. Ophthalmological and audiometric examinations were normal.

Ventricular extrasystoles disappeared and QT distance normalized soon after intravenous Ca administration. Parenteral Ca-gluconate was given for three days (10 mL/dose; 4, 3 and 2 doses in consecutive days). Ca-acetate, 3x250 mg/dose PO, was also added on the second day. Serum Mg concentration improved over three days (1.3, 1.5 and 1.8 mg/dL, respectively) without Mg administration. 1,25-dihydroxyvitamin D3, in a dose of 1 μg/day and 40 mg/kg/day elementary Ca together with a low-phosphorus diet, were started. The characteristic phenotypic stigmata and laboratory results led us to consider a diagnosis of PHP type 1a. Echocardiogram, 24-hour Holter monitoring, electroencephalogram, and kidney ultrasonography results became all normal after the normalization of Ca and Mg levels. 

## DISCUSSION

While tetany or seizures secondary to hypocalcemia are common presentations in patients with PHP, cases with hypocalcemia-induced cardiac symptoms are quite rare and, to our knowledge, only two patients presenting with cardiac syncope have been reported (3,4). The present case is the first one of hypocalcemia-induced ventricular arrhythmia in a patient with PHP. While hypocalcemia-induced QTc prolongation has been proposed as the possible pathogenesis of the syncope in the previous patients, no arrhythmias were reported (3,4). Despite having no syncope, the present case may suggest that hypocalcemia results in QTc prolongation, ventricular arrhythmia, and hence, syncope in patients with PHP. Hypomagnesemia is another striking feature of both the present and the other two cases (3,4). It is known that hypomagnesemia or hypocalcemia can cause prolongation in QT intervals and arrhythmia (5). However, although hypomagnesemia has been reported in PHP (3,4,6), the pathogenesis and clinical significance of these findings have not yet been fully elucidated. Hypomagnesemia is often associated with hypocalcemia due to both lower PTH secretion and end-organ resistance. An increase in serum Mg level, paralleling the increase in serum Ca, was also noted in our patient (7,8,9). Our patient also had high CK and LDH levels. Studies have demonstrated that chronic hypocalcemia or hypomagnesemia may cause muscle degeneration (10,11).

The findings in the present case support previous reports and demonstrate that hypomagnesemia may make patients with PHP more susceptible to hypocalcemic complications such as cardiac arrhythmias and myopathic changes.
